# Case Series: Severe cholangitis as an immune checkpoint inhibitor induced adverse event in four cases: management and treatment options including tocilizumab and ursodeoxycholic acid

**DOI:** 10.3389/fimmu.2026.1929147

**Published:** 2026-10-27

**Authors:** Darina Kohoutova, Joshua Shur, Monica Terlizzo, Robert Goldin, Matthew Foxton, Nicholas Powell, Douglas Macdonald, Naureen Starling, Sheela Rao, Kevin Joseph Harrington, Kate Young, Sanjay Popat, Nicos Fotiadis, Edward W. Johnston, Sameer Zar, Daniel Levine, Maroua Hafi, Pablo Jimenez-Labaig, Abu Taher, David C. Cunningham

**Affiliations:** 1 The Royal Marsden Hospital NHS Foundation Trust, London, United Kingdom; 2 Imperial College Healthcare NHS Trust, London, United Kingdom; 3 Royal Free Hospital NHS Foundation Trust, London, United Kingdom

**Keywords:** case report, cholangitis, immune check-point inhibitor, liver biopsy, tocilizumab, ursodeoxycholic acid

## Abstract

**Introduction:**

Immune checkpoint inhibitors (ICIs) have revolutionized treatment of multiple malignancies, nevertheless, their clinical use can be associated with adverse events (AE). ICI-induced cholangitis (ICI-C) belongs to rare AEs. Poor response to glucocorticosteroids (GCS) is observed, deaths related to ICI-C were reported.

**Main findings and therapeutic interventions:**

We share four detailed cases (two males and two females) of severe ICI-C (severity assessed based on DILIN criteria). All patients were jaundiced (bilirubin >90 µmol/L), one had cholestatic liver injury, two had mixed liver injury and one had a rather hepatocellular liver injury based on R factor in DILI. Three were treated with programmed cell death protein (anti-PD1) or ligand 1 antibodies, one with anti-PD1 and cytotoxic T-lymphocyte-associated protein-4 antibodies. Onset of liver injury was observed during treatment (2), three weeks after last infusion (1) and four months after last treatment (1). Laboratory tests excluded infections, malignant infiltration and autoimmune profile was typically negative. Another drug induced injury was excluded. MRCP and EUS were used as imaging methods of choice. A liver biopsy was organized due to absent response to corticosteroids and second line immunosupressive medication, as cholangitis was suspected. Histology was confirmatory for ICI-C in all cases. Predominant infiltration by CD8 positive lymphocytes was shown. Treatment with tocilizumab, anti-IL-6 receptor antibody, and ursodeoxycholic acid were used in three cases.

**Outcomes:**

Three patients with ICI-C confirmed histologically received tocilizumab and ursodeoxycholic acid with an excellent effect. Decrease in bilirubin was observed within 72 hours. Significant improvement/normalization of liver enzymes followed. No side effects of tocilizumab were reported. Patient who did not receive tocilizumab (maximum bilirubin 349 µmol/L, encephalopathy; cholestatic liver injury – R factor 0.8) died.

**Conclusion:**

We advocate for urgent work-up including early liver biopsy the more that immune mediated liver injury from checkpoint inhibitors can cause different laboratory pictures including mixed ones as well as hepatocellular injury (if assessed by DILIN) and still ICI-C can be confirmed on histology. Treatment with tocilizumab, in combination with ursodeoxycholic acid, can be considered in severe cases of ICI-C.

## 1 Introduction

Immune checkpoint inhibitors (ICIs) have revolutionized treatment of multiple malignancies, achieving long-term disease control and, in some cases, even cure; nevertheless, their clinical use can be associated with adverse events (AE) including severe ones (1, 2). ICI-related hepatitis (ICI-H, associated typically with elevation of ALT and AST) belongs to the category of less common adverse events and is observed in 5%-10% (1%-2% grade 3) of patients treated with ICI monotherapy and in 25%-30% (15% grade 3) with anti-PD-(L)1 (programmed cell death ligand 1 antibodies) and anti-CTLA-4 (cytotoxic T-lymphocyte-associated protein-4 antibodies) combination therapy (3, 4). ICI-induced cholangitis (ICI-C) belongs to the group of rare AE and elevation of ALP and GGT is typically more prominent when compared to ALT and AST (3). While ICI-H usually resolves within 2–4 weeks if treated appropriately, ICI-C responds to treatment slowly, only 9% of patients achieve complete normalization of biliary enzymes (5), moderate to poor response to steroid therapy is observed (6, 7) and deaths related to ICI-C have been reported (8, 9). Terminologically, both, ICI-H and ICI-C, belong to ILICI, Immune-medicated Liver Injury from Checkpoint Inhibitors (10).

We share four consecutive detailed cases of severe (2) and moderate – severe (2) ICI-C (in those where tocilizumab was considered due to severity of the AE) and provide guidance for patients with a possible ICI-C. DILIN (Drug-Induced Liver Injury Network) criteria have been used to grade severity of ILICI. We advocate for: 1) an urgent work-up (laboratory, imaging); 2) an early liver biopsy if MRCP/EUS show no or very mild biliary pathology and 3) if ICI-C is confirmed, we recommend prompt commencement of immunosupressive therapy. Liver biopsy can confirm ICI-C even if the laboratory picture is not purely cholestatic based on DILIN criteria. Absent/poor response to glucocorticosteroids needs to be taken into account. Treatment with tocilizumab, an anti-IL-6 receptor antibody, and UDCA (ursodeoxycholic acid) may be the preferred option in severe ICI-C.

## 2 Case report 1

A 65-year-old man was diagnosed with a metastatic adenocarcinoma of the gastro-esophageal junction, stage T2N3M1, HER-2 negative, MMRp, PD-L1 negative. He was treated with CAPOX, subsequently he was enrolled into a combination trial including PD-L1 inhibition with 2-weekly atezolizumab and a WNT inhibitor. Twelve months later he was diagnosed with an acute pancreatitis (elevated pancreatic enzymes, epigastric pain, imaging compatible with acute pancreatitis) induced by ICI and treatment with atezolizumab was discontinued. Serum IgG4 was within normal ranges. Acute pancreatitis was refractory to treatment with glucocorticosteroids (given orally, then intravenously 1mg/kg, then orally for duration of 2 months) as well as to mycophenolate mofetil (MMF 1g BD, added to treatment for another 1 month), but responded well to tacrolimus (4.5mg BD orally). At the time of pancreatic enzyme normalization, four months after the last administration of atezolizumab, a sudden elevation of ALT, ALP and bilirubin from normal serum levels was observed. CT did not show any intrahepatic or extrahepatic biliary dilatation. EUS confirmed thickened wall of a non-dilated common bile duct (**Figure 1**), compatible with an ICI-induced cholangitis. Liver biopsy confirmed ICI-C (**Figure 2**). ANCA antibodies were negative. UDCA was introduced (15mg/kg), corticosteroids were increased to a dose of 10mg/kg intravenously 8 days prior to the death. Treatment with tocilizumab was approved, but the patient deteriorated within 24 hours, encephalopathy was present, and died the evening before the planned administration of tocilizumab, four weeks after the onset of cholestasis. The serum levels on the day of patient´s death were: ALT: 428 U/L (9x ULN); ALP: 1,328 U/L (11x ULN); total serum bilirubin: 349 umol/L (16x ULN). R ratio in DILI was 0.8 confirming cholestatic pattern of liver injury.

**Figure 1:**
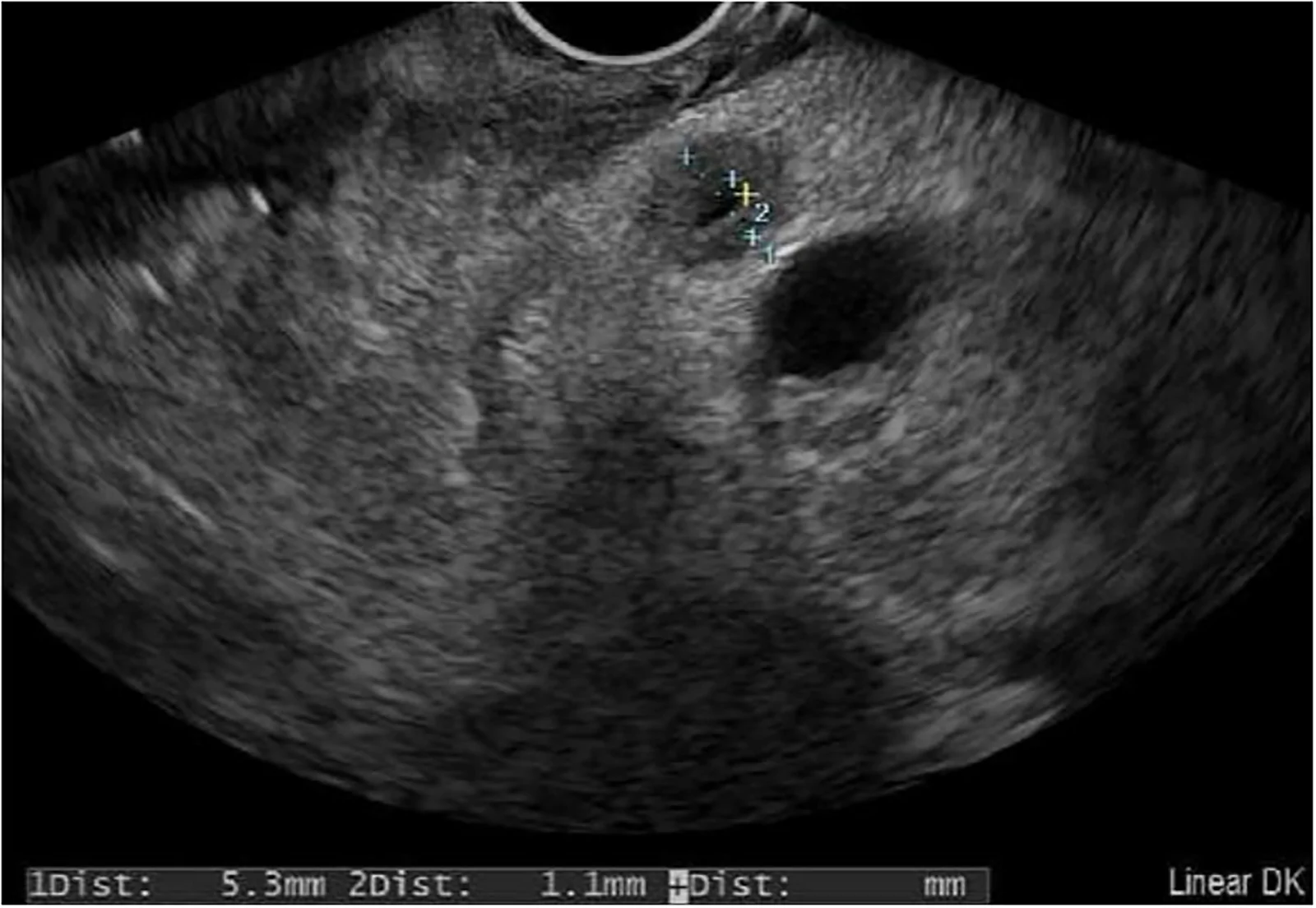
Endosonography finding: thickened wall (2mm) of a non-dilated common bile duct (5.3mm).

**Figure 2:**
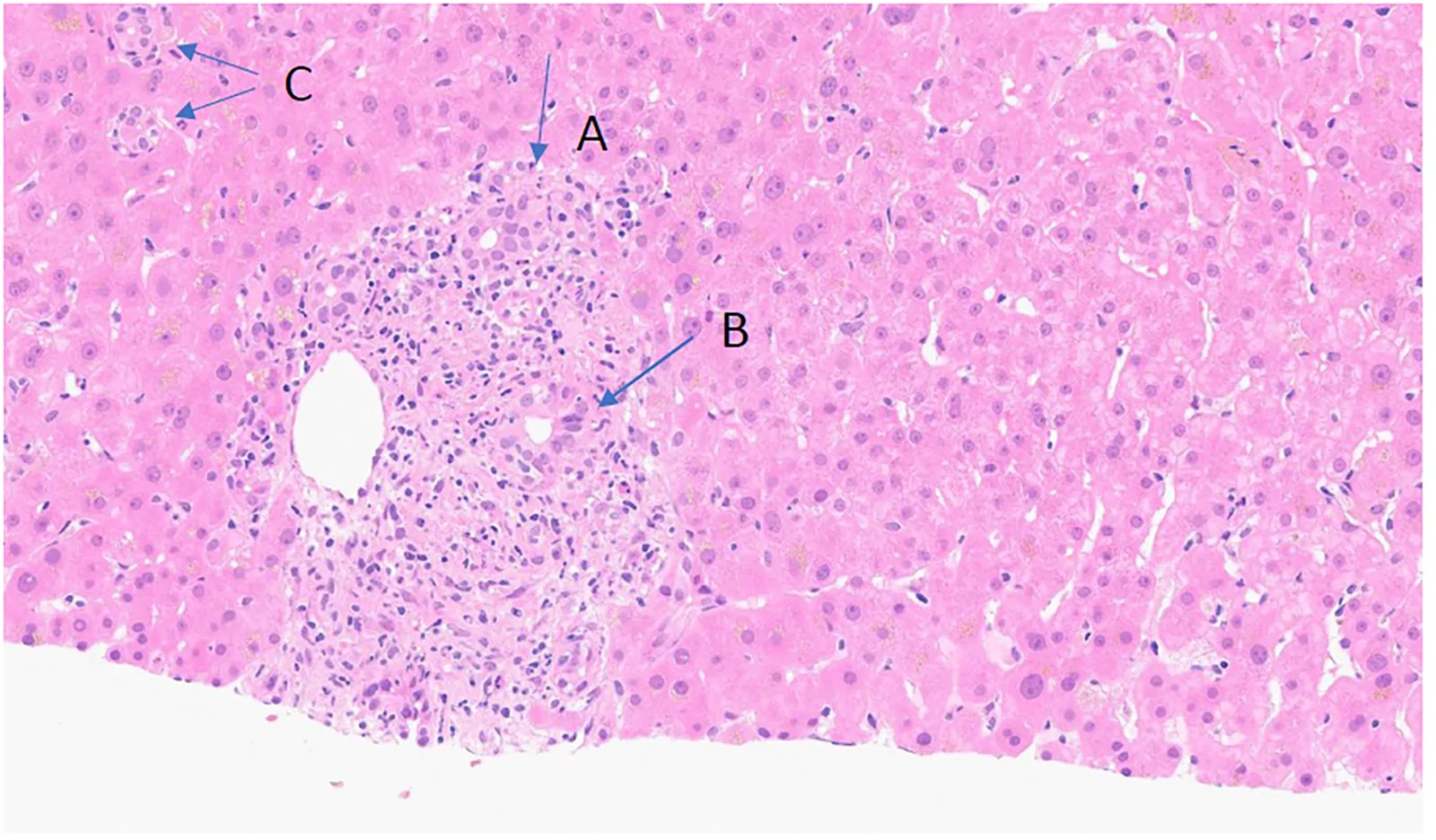
Liver biopsy, Hematoxylin-eosin staining; original magnification: 200 **(A)** Moderate portal and periportal inflammation composed of mainly lymphocytes and macrophages. **(B)** Inflammatory bile duct damage with vacuolization of the bile duct epithelium with intra-epithelial lymphocytes. **(C)** Spotty necrosis.

## 3 Case report 2

A 53-year-old female patient was diagnosed with adenocarcinoma of the esophagus stage T3N3M0, MMRp, HER-2 negative, PD-L1: 17, Claudin 18.2 negative. She underwent perioperative chemotherapy (FLOT) and esophagectomy (including left hemithyroidectomy, supraclavicular fossa nodal dissection and left neck node dissection). Subsequently, for recurrence of cancer, she was offered chemotherapy (CAPOX), ICI (pembrolizumab) and radiotherapy (right supraclavicular fossa node, paraesophageal recurrence and retrocrural node). Maintenance with pembrolizumab followed (3-weekly, 12 months). A sudden elevation of serum bilirubin and liver enzymes was observed within two weeks after the last administration of pembrolizumab and treatment with ICI was discontinued. Findings on MRCP were normal, EUS was not possible due to a tight malignant esophageal stricture. Viral hepatitis (A, B, C & E, CMV and EBV) and autoimmune hepatitis screen were negative. She was treated with intravenous corticosteroids (methylprednisolone 1mg/kg) and UDCA 10mg/kg for 7 days; dose of methyprednisolone was increased to 2mg/kg on day 8 and mycophenolate mofetil 500mg BD was added on day 11 to no avail with bilirubin still increasing. ICI-C was in differential diagnosis and therefore a liver biopsy followed. Histology was indeed compatible with ICI-C: it confirmed presence of cholestasis, fibrosis and spotty necrosis (**Supplementary Figures S1**, **S2**). Liver enzymes and bilirubin deteriorated from normal serum levels within four weeks and reached the following values: ALT: 381 U/L (11x ULN); ALP: 211 U/L (2x ULN); bilirubin 253 umol/L (12x ULN). The R ratio in DILI was 6.5, typical for hepatocellular liver injury. Yet, histology from liver biopsy showed cholestatic liver injury convincingly. Due to absence of response to any previous immunosupressive medication, therapy with tocilizumab (8 mg/kg, administered intravenously) followed on day 17. A quick decrease in serum bilirubin was observed after the tocilizumab treatment: (serum level: 161 umol/L (7x ULN) at 72 hours, 48 umol/L (2x ULN) at 1 month). Serum ALP normalized fully (126 U/L) within 13 days after tocilizumab administration. A delayed effect of tocilizumab towards ALT was observed: serum ALT level normalized (30 U/L) in 2 months. Treatment with corticosteroids, mycophenolate mofetil and ursodeoxycholic acid still continued during this period.

## 4 Case report 3

A 78-year-old female patient was diagnosed with a squamous cell carcinoma of the upper lip, stage pT3N0M0; PD-L1> 1. She underwent an excision and reconstruction, yet suspicious mediastinal lymph nodes and progressive disease of the lip were observed three months later. Palliative radiotherapy at the upper lip followed and systemic treatment with pembrolizumab and evorpacept (ALX-148), an antibody targeting CD47 to block “don’t-eat-me” signals and activate immune-related tumoural phagocytosis, was introduced in the context of a clinical trial. Three weeks after the very last administration of pembrolizumab (two years of ICI had been completed), a sudden elevation of liver tests including ALT and ALP accompanied by elevated bilirubin were observed. Viral hepatitis (A, B, C & E, CMV and EBV) and autoimmune hepatitis screen were negative. The maximum serum levels were as follows: bilirubin: 54 umol/L (2x ULN); ALP: 494 U/L (4x ULN) and ALT: 596 U/L (17x ULN). The R ratio in DILI was 4.3 showing a mixed liver injury at that point. The condition was treated with oral & intravenous corticosteroids as well as mycophenolate mofetil successfully. Three months later, medication was weaned off, unfortunately recurrence of bilirubin and liver enzymes elevation was observed one month later. The maximum serum levels were at this point: bilirubin: 95 umol/L (4x ULN); ALP: 482 U/L (4x ULN) and ALT: 435 U/L (12x ULN). The R ratio in DILI was 3.2 being compatible with a mixed liver injury. MRCP showed irregularity of intrahepatic bile ducts (**Supplementary Figure S3**); common bile duct was smooth and thin-walled. Serum IgG4 was within normal limits and so was ANCA. Patient was treated for 4 weeks in total with ursodeoxycholic acid, corticosteroids (1mg/kg for 4 days, then 2mg/kg for almost 4 weeks) and mycophenolate mofetil (1g BD) which lead to a temporary improvement in liver tests and bilirubin levels. Yet, another peak of further elevation of bilirubin, ALP and ALT was observed: bilirubin: 57 umol/L (3x ULN); ALP: 271 U/L (2x ULN) and ALT: 324 U/L (9x ULN). The R ratio in DILI was 4.3 being compatible with a mixed liver injury. A liver biopsy was organized. Histology confirmed ICI-C with predominant CD8+ lymphocytes infiltration and new ductulae formation (**Supplementary Figure S4**; **Figure 3**). Two doses of tocilizumab 8 mg/kg were administered 5 days apart. Serum bilirubin normalized fully within 11 days (20 umol/L) as well as ALP normalized fully within 10 days (115 U/L); ALT decreased to 1x ULN (47 U/L) within 1 month. The fourth, last deterioration of liver tests was explained by reintroducing the treatment with statin (Atorvastatine, 40 mg daily). The peak serum levels during this deterioration were: bilirubin: 57 umol/L (3x ULN); ALP: 229 U/L (2x ULN) and ALT: 599 U/L (17x ULN). The R ratio in DILI was 9.4, much higher, being compatible with a hepatocellular (drug induced) liver injury. Patient had been on statin for one year before the onset of ICI-C, however medication was stopped since cholestatic liver injury was first observed. Atorvastatin was recommenced three days prior to the onset of the last bilirubin deterioration and has therefore been discontinued permanently since. Another treatment with tocilizumab was not needed. Further immunosuppressive treatment with corticosteroids, mycophenolate mofetil and therapy with ursodeoxycholic acid have continued.

**Figure 3:**
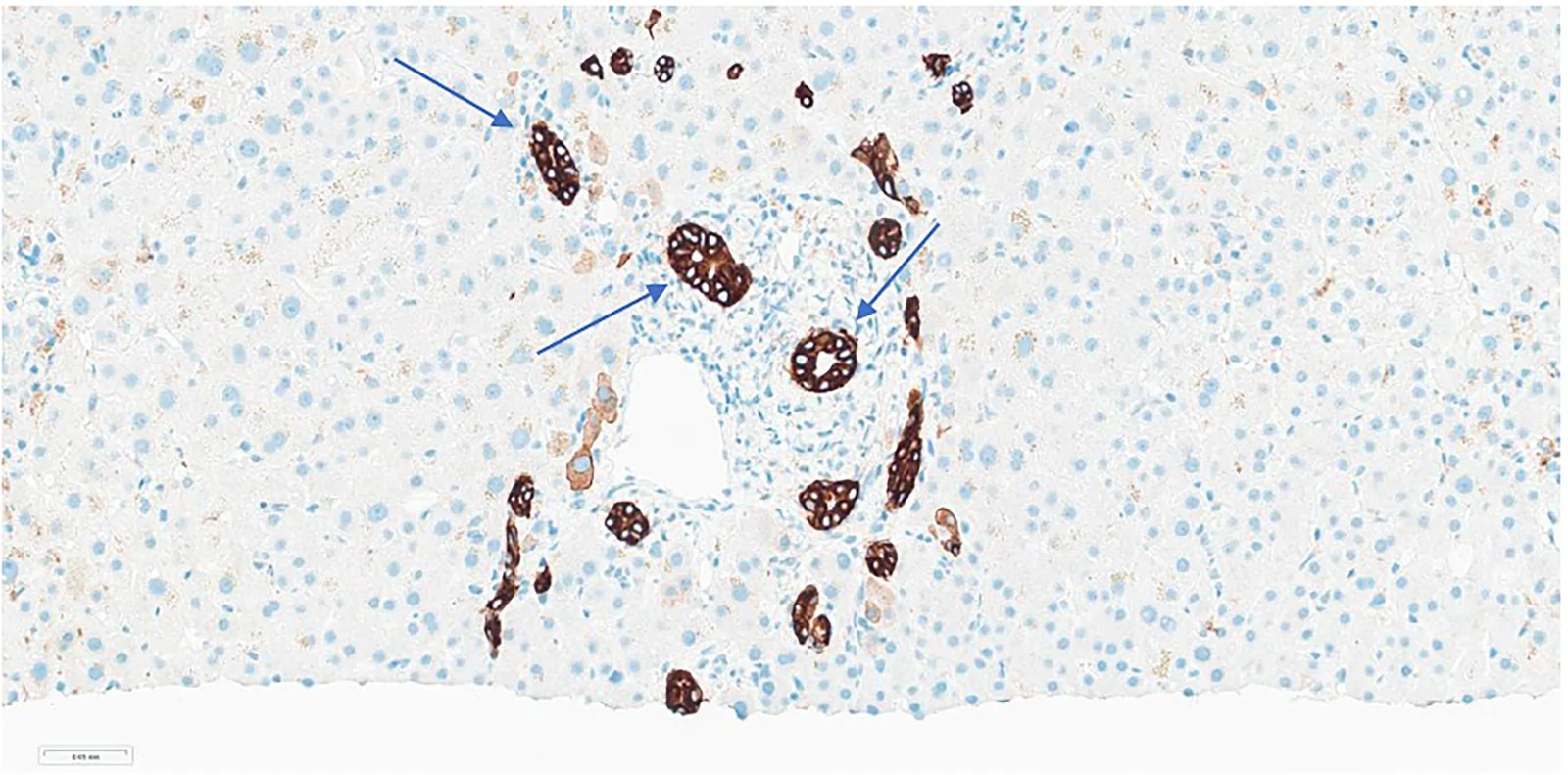
Liver biopsy, immunohistochemistry using cytokeratin 7; original magnification: 40. New ductulae formation (arrow).

## 5 Case report 4

A 69-year-old man was diagnosed with metastatic melanoma BRAF V600E IHC negative, NGS V600R and K610E mutation. Combination of ipilimumab and nivolumab was commenced shortly after the diagnosis. After the first cycle, he developed grade I elevation of ALT and ALP (levels around three times the upper limit of normal) which deteriorated further after cycle two. Immunotherapy was, therefore, discontinued and methylprednisolone 1mg/kg was started. EUS followed and this identified a dilated common bile duct (maximum diameter: 13 mm) with no obstruction. Despite treatment with methylprednisolone (1mg/kg intravenously for 4 days, then 14 days orally, dose being 65 mg first, then reduced to 50 mg daily, total duration of steroid treatment was 18 days before switching back to the intravenous form), liver tests deteriorated further and rise in bilirubin was observed. MRCP showed a possible focus of material in the distal CBD (common bile duct), therefore ERCP followed. There was non-obstructive sludge in the CBD and a short stricture at the distal CBD. Sphincterotomy was performed, sludge was extracted and a plastic stent was inserted. Cytology from the stricture was benign. Yet, bilirubin continued to rise, MRCP and CT images were suspicous of cholangitis (**Supplementary Figures S5a, b**) and, therefore, a trans-jugular liver biopsy was organized. This showed active chronic hepatitis with bile duct damage, vacuolization of the bile duct epithelium, newly formed ductule structures, spotty necrosis of hepatocytes and hepatocellular cholestasis. ANCA were negative and IgG4 mildly elevated (at 2.22g/L). IgG4 positive cells were not present on histopathology. Liver tests deteriorated further and reached the following maximum serum levels: ALT: 1664 U/L (33x ULN), ALP: 1415 U/L (11x ULN), bilirubin: 169 umol/L (8x ULN). R ratio for DILI was 3.0 compatible with mixed pattern of liver injury. Methylprednisolone 2mg/kg was given for 3 days, Mycophenolate (1g BD) was added on day 3 and treatment with tocilizumab (8mg/kg) followed on day four. Ursodeoxycholic acid (250 mg BD) was commenced. Serum bilirubin level decreased immediately and its almost complete normalization was observed within 11 days (27 umol/L; 1x ULN). Decrease in serum ALT and ALP were also observed over the following days. Complete normalization of ALT was seen in 5 weeks (35 U/L). ALP dropped to 300 U/L within 2 months and has been oscillating between 2x ULN – 3x ULN since. Therapy with mycophenolate mofetil was stopped seven months after the administration of tocilizumab, treatment with UDCA has continued.

Based on past medical (drug) history, laboratory findings and imaging, DILI unrelated to ICI could be excluded in all cases (with the exception of the last deterioration of liver tests caused by medication with statins in case 3). No patient had metastatic involvement of the liver.

Based on DILIN criteria, all patients had severe or moderate-severe ICI. All individuals were admitted due to immune checkpoint inhibitor induced liver injury. All patients had raised ALT and ALP levels and all had bilirubin >90 µmol/L. The first patient had INR 1.5 eight days before he died (with no further deterioration observed) as well as encephalopathy. The third patient had prolonged jaundice and symptoms beyond 3 months.

## 6 Discussion

### 6.1 Classification of ICI-C

ICI-C can be classified into three subtypes depending on the anatomy and the size of involved bile ducts (5, 11): 1) small-ducts type (intrahepatic small bile ducts); 2) large-ducts type (intrahepatic large bile ducts (= from segmental intrahepatic ducts and above and extrahepatic bile ducts); 3) mixed type.

### 6.2 Biochemistry

Predominant elevation of cholestatic liver enzymes (ALP and GGT) is usually present (6). Yet, mixed cholestatic and hepatocellular pattern can also be observed. Conjugated hyperbilirubinemia has been present in severe ICI-C cases based on our experience.

#### 6.2.1 ALP

Pi et al. (5) described that serum ALP in individuals with large-ducts subtype is significantly higher than in small-ducts subtype (1683 vs 678 IU/L; p=0.02). No difference in ALP level was observed between mixed and large-ducts subtype and between mixed and small-ducts subtype. This was replicated in our cases (first patient: mixed type, maximum ALP: 1455 U/L; second patient: small ducts subtype, maximum ALP: 227 U/L; MRCP with normal findings, cholangitis confirmed on liver biopsy; third patient: small ducts subtype, maximum ALP: 494 U/L; MRCP with mild intrahepatic ICI-C; fourth patient: mixed type, maximum ALP: 1415 U/L). The prognosis and treatment do not seem to depend on the level of duct involvement, however more data is required.

### 6.3 Absence of autoimmune serological markers

Elevation of serum IgG4 is not observed in ICI-C (7, 8, 12, 13). Further, effectiveness of steroid therapy belongs to diagnostic criteria of IgG4-related sclerosing cholangitis, whereas moderate to poor response to glucocorticosteroids has been described in ICI-C (7, 14, 15).

Serum autoantibodies are not typically detected in ICI-C (7, 16). This can provide guidance in differential diagnosis with primary sclerosing cholangitis (ANCA antibodies) (17); autoimmune hepatitis (non-organ-specific autoantibodies: ANA and ASMA; organ-specific antibodies: anti-LKM1 and anti-SLA/LP) (18); and primary biliary cholangitis (antimitochondrial antibodies) (19).

### 6.4 ICI-C: which immunotherapy and when

It has been documented that pembrolizumab and nivolumab, anti-PD1s, were responsible for the majority of reported cases of ICI-C (5, 20, 21). However, other ICIs including ipilimumab, an anti-CTLA-4, avelumab, atezolizumab and durvalumab, anti-PD-L1s, as well as ICI combinations have also been associated with the onset of ICI-C (5, 22, 23).

The number of ICI cycles until the onset of ICI-C was reported to be different in small-ducts type (median: 2.5; 1-13), mixed type (median: 5; 3-27) and large-ducts type (median: 7; 1-25) (5).

### 6.5 ICI-C: male predisposition possible

It is known that females are more susceptible to autoimmune diseases (24) as well as development of immunotherapy-related AEs (25). Unger et al. analysed the incidence of severe AEs in those treated with immunotherapy (n=2,319) and confirmed that women had a 49% increased risk compared to men (26).

Yet, Onoyama et al. described thirty-one PD-1 inhibitor-related sclerosing cholangitis cases with a male to female ratio of 21:10 (27). Subsequently, Pi et al. confirmed in his systematic review (5) that ICI-C is indeed not more commonly observed in women (35 men and 18 women with ICI-C identified). Retrospective analysis of ICI-C cases reported to the French pharmacovigilance system acknowledged equal distribution between males and females (24 cases vs 24 cases) (28).

### 6.6 Imaging

MRCP, MRI liver with contrast and EUS should be the preferred imaging modalities if ICI-C is suspected. In patients with small ducts-type, no or minimal pathology is observed (**Supplementary Figure S3**). In mixed type and large-duct types, the following pathologies can be seen: intrahepatic and/or extrahepatic biliary dilatation with no obvious biliary obstruction, stricturing and irregularity of intrahepatic biliary ducts, biliary duct wall thickening and periductal enhancement (**Figures 1**, **4**). Findings can be segmental or diffuse (5, 7, 8, 12, 13, 16, 29, 30).

**Figure 4:**
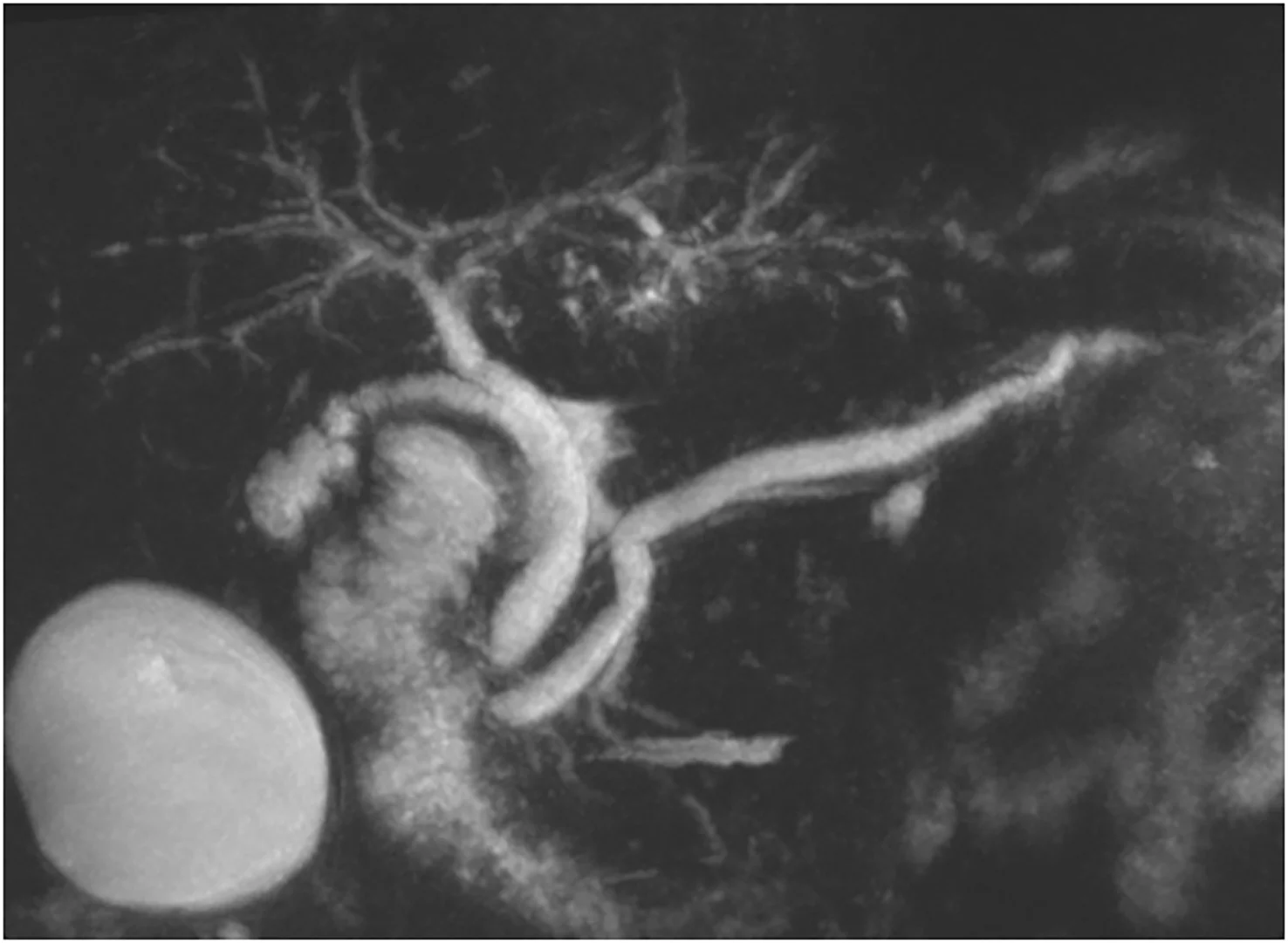
MRCP MIP. Bile duct wall thickening, irregular intrahepatic dilatation.

PET CT is a modality often used for surveillance of patients treated with ICIs. Due to its sensitivity, uptake in the biliary tree, including in the gallbladder, can be noticed even without any associated laboratory findings and/or clinical symptoms (**Supplementary Figures S6a, b**). These patients need to remain monitored closely.

### 6.7 ERCP

There is no role for an ERCP unless there is a co-incidental pathology which would require such an intervention (our case 4). ERCP can be associated with a high risk of complications, including introduction of an infection/biliary sepsis. Therefore, all patients with ICI-C in whom ERCP is being considered for a co-incidental biliary pathology should be discussed within a multidisciplinary team.

### 6.8 Liver biopsy, histology, immunohistochemistry

Liver biopsy plays a central role in establishing the diagnosis of ICI-related cholangitis, particularly in patients with a cholestatic liver injury, but also in patients with mixed liver injury and even in those with a rather hepatocellular picture (case 2) where non-invasive investigations are non-diagnostic. Biopsy can distinguish ICI-related cholangitis from alternative causes, including another drug-induced liver injury, autoimmune hepatobiliary disease, or malignant infiltration. Histological assessment can provide important diagnostic confirmation and may help to guide subsequent immunosuppressive therapy. DILI criteria and R ratio can help to guide in ILICI, however ICI-C needs to be excluded on liver biopsy even if hepatocellular liver injury is rather present based on R factor in DILI.

Typical findings on histology can mimic primary sclerosing cholangitis including ductopenia (vanishing bile duct syndrome), formation of new ductules, periductal fibrosis (“onion-like skin”) and cholestasis. Periportal inflammation composed mainly of CD8 positive lymphocytes as well as spotty necrosis can be seen. Intraepithelial lymphocytes (primary biliary cholangitis-like) as well as scattered lobular lymphocytes can be observed (5, 13, 16, 24, 29, 31).

### 6.9 Treatment options

Resistance to immunosupressive treatment as well as life-threatening course of ICI-C have been reported repeatedly (7–9). We, therefore, advocate for prompt therapeutic intervention which should include consideration of tocilizumab, especially in those with a rapid deterioration of cholestastic liver enzymes and/or in those who have conjugated hyperbilirubinemia present already. UDCA should always be considered, too.

#### 6.9.1 Cessation of immunotherapy

This should be recommended if ALP and/or GGT > 3x upper limit of normal values [similar to ICI-H (3)].

#### 6.9.2 Corticosteroids

Response to corticosteroid administration is not only slow (this can be explained by the long half-life of anti-PD-1; e.g. 17–25 days for nivolumab) (31), but, even more importantly, the overall complete response rate to corticosteroids was found to be low, 11.5%, in program cell death inhibitor-related sclerosing cholangitis (27). The majority of ICI-C patients will have moderate or poor response to corticosteroids, including to high doses (500-1000mg/day) (8, 27).

We would recommend commencing corticosteroids at a dose of 0.5-1mg/kg/day if ALP and/or GGT > 3x upper limit of normal values; however, we emphasize the need for a further immunosupressive treatment if no major rapid improvement in cholestatic liver injury to intravenous glucocorticosteroids is observed within 3 days.

#### 6.9.3 Ursodeoxycholic acid, UDCA

UDCA, the cornerstone of primary biliary cholangitis (PBC) treatment since its approval in 1994, has multiple beneficial effects. UDCA is a hydrophilic bile acid which replaces more hydrophobic and toxic bile acids. It has a choleretic effect, promotes bile secretion, improves liver function and alleviates cholestasis. Further, UDCA acts as a cytoprotective, anti-inflammatory and immunomodulatory agent (32). Importantly, UDCA exerts anti-apopotic properties (33). Chronic medication with UDCA in PBC has been shown to reduce aberrant hepatocyte HLA class I expression, which then may lead to decreased cytotoxic T-cell dependent lobular necrosis (34). Therefore, this immune-mediated protective effect of UDCA could play an important role in ICI-C where cytotoxic CD8 lymphocytes have been documented to be the predominant entity in liver biopsies.

European Society for Medical Oncology recommends that patients with ICI-C should be treated with UDCA and prednisone/budenoside (3). Optimal dose, duration and exact efficacy of UDCA is yet to be established (24).

Pi et al. reported in his systematic review, that 20/53 patients were treated with UDCA. After the withdrawal of glucocorticosteroids in some patients, UDCA was continued long-term and the decline of ALP was still shown (5). This is in agreement with the observation of Li et al. (16): liver tests deteriorated after an initial improvement with glucocorticosteroids in a patient with pembrolizumab-induced cholangitis. After UDCA (500 mg BD) had been added, complete normalization of liver tests was confirmed. Fang et al. found, that 22 out of 25 patients with cholangitis, who were commenced on UDCA, improved (29). A total of 27% patients with ICI-C reported to the French database of pharmacovigilance received UDCA. The authors acknowledge that UDCA should be recommended as the first-line therapy with or without glucocorticosteroids (28).

We would recommend a dose of 15 mg/kg/day in every patient with ICI-C in the absence of any contraindications. Long-term medication should be opted for, safety profile being favorable.

#### 6.9.4 Tocilizumab

The use of tocilizumab, an anti-IL-6 receptor (anti-interleukin-6 R) agent, has been reported in treatment of ICI-C in case reports or small series so far. Anti-IL-6R, similarly to other second-line immunosuppressive medications, is recommended only for steroid-refractory immunotherapy related AEs (35–37). IL-6 is a proinflammatory cytokine which is produced early during the process of inflammation (38). Tocilizumab, anti-IL-6R, inhibits signaling downstream of interferon-gamma and other Janus kinase-dependent cytokines (37). Faak et al. described five cases of ICI-C treated with tocilizumab: three patients received one infusion (8 mg/kg), two received more infusions 4 weeks apart. An improvement was observed in four out of five patients within 12 days. All patients had failed to respond to corticosteroid therapy initially (35). Pi et al. (5) included in their systematic review four patients who were reported to receive tocilizumab: UDCA and tocilizumab stabilized ICI-C in a female patient after several lines of treatments had failed (36). Moi et al. described three patients (two males and one female) with steroid-refractory ICI-C who received a personalized treatment with tocilizumab on the basis of their liver biopsy’s predominant immune infiltrate (T lymphocytes) and cytokine profile (high proinflammatory cytokines including INF-γ and INF- γ induced chemokines). All achieved persistent clinical and biological remission (37).

Currently, limited data on the efficacy of second-line therapeutic options in ICI-C is available (24, 37). Tocilizumab seems to be an effective steroid-sparing agent for treatment of immune-related AEs (35, 39, 40). Daoudlarian et al. confirmed efficacy of tocilizumab in patients experiencing high grade immune related cytokine release syndrome (41); tocilizumab has also shown benefit in treating ICI-associated arthritis and prevented its relapse during ICI rechallenge (42). Yet, further prospective data on efficacy, safety of tocilizumab and its impact on cancer outcomes in patients with ICI-C is required.

Tocilizumab comes with an MHRA warning in patients with active hepatic disease or hepatic impairment. Its use has been associated with a rare risk of serious liver injury, including cases requiring transplantation (43). Thus an appropriate informed consent has to be obtained. We have not observed any side effects caused by tocilizumab in our patients.

#### 6.9.5 MMF, tacrolimus, azathioprine

If there is no laboratory response to intravenous corticosteroids, other second-line immunosuppressive therapies should be considered. These include mycophenolate mofetil (1000 mg BD), tacrolimus (0.06 mg/kg BD, aiming for trough level of 5–12 ng/mL; checking trough levels 3 days after starting the medication and then twice-weekly until reaching a steady state), and azathioprine (50 mg/day typically; activity of thiopurine methyltransferase should be checked prior to administration of azathioprine) (5, 7, 13, 29).

Severity of the ICI-C and the rate of deterioration in laboratory findings needs to be considered. Clinical effect of mycophenolate mofetil is observed in 4–6 weeks (44), with the maximum clinical benefit being present within 5 months (45). Clinical response to tacrolimus is seen typically within days depending on the route of administration (46). The time to onset of action for azathiprine is 8–12 weeks (47).

In agreement with Haanen et al. (3), we do not recommend treatment with infliximab in patients with ICI-C.

Table 1 summarizes management of ICI-C, **Supplementary Figure S7**.

## 7 Conclusion

ICI-C is a rare AE. Albeit spontaneous improvement has been observed in mild ICI-C including among our patients, this can be a life-threatening and fatal AE. We recommend a multidisciplinary collaboration between gastroenterologists, oncologists, radiologists and histopathologists. Typically, cholestatic liver injury is observed in laboratory tests. Hyperbilirubinemia is associated with a more severe course. Albeit, mixed (cholestatic and hepatocellular) picture and exceptionally hepatocellular picture can be present in biochemistry, ICI-C can be confirmed histologically. Therefore, imaging (MRCP, EUS) and liver biopsy are crucial in establishing the diagnosis of ICI-C promptly. Response to treatment with glucocorticosteroids is moderate to poor; therefore, other immunosuppressive medications should be considered urgently. Mycophenolate mofetil, azathioprine and tacrolimus can be used. In patients with a rapid laboratory deterioration, tocilizumab, an anti-IL-6R agent, can be used with success, as observed in our patients. UDCA is recommended to be added early on, taking into account its favorable safety profile and its multiple beneficial properties.

## Data Availability

The original contributions presented in the study are included in the article/**Supplementary Material**. Further inquiries can be directed to the corresponding author.
