# Supplementary material for: Case Series: Severe cholangitis as an immune checkpoint inhibitor induced adverse event in four cases: management and treatment options including tocilizumab and ursodeoxycholic acid
Source: Front Immunol. 2026 Oct 27;17:1929147. doi: 10.3389/fimmu.2026.1929147 (PMC13756901; doi:10.3389/fimmu.2026.1929147)

Figure S2

Liver biopsy, Haematoxyllin-eosin staining; original magnification: 200

Spotty necrosis (asterisk) and cholestasis (arrow)

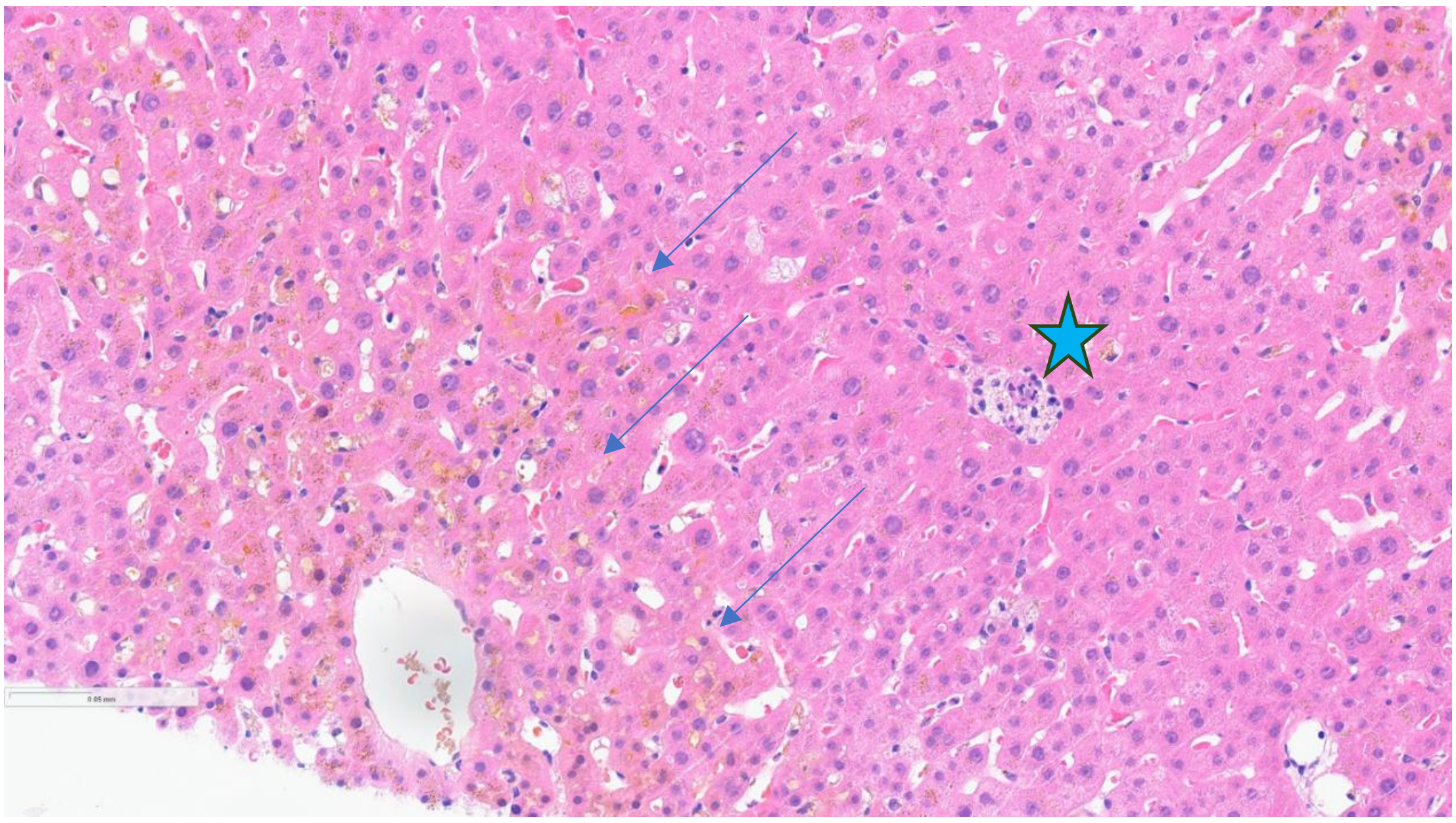

Supplement: Supplementary file 2 [file SupplementaryFile2.pdf]
