# Supplementary material for: Case Series: Severe cholangitis as an immune checkpoint inhibitor induced adverse event in four cases: management and treatment options including tocilizumab and ursodeoxycholic acid
Source: Front Immunol. 2026 Oct 27;17:1929147. doi: 10.3389/fimmu.2026.1929147 (PMC13756901; doi:10.3389/fimmu.2026.1929147)

Figure S3

MRCP volumetric MIP (maximum intensity projection) demonstrating stricturing and irregular dilation of the intrahepatic bile ducts (circled) consistent with a cholangitis.

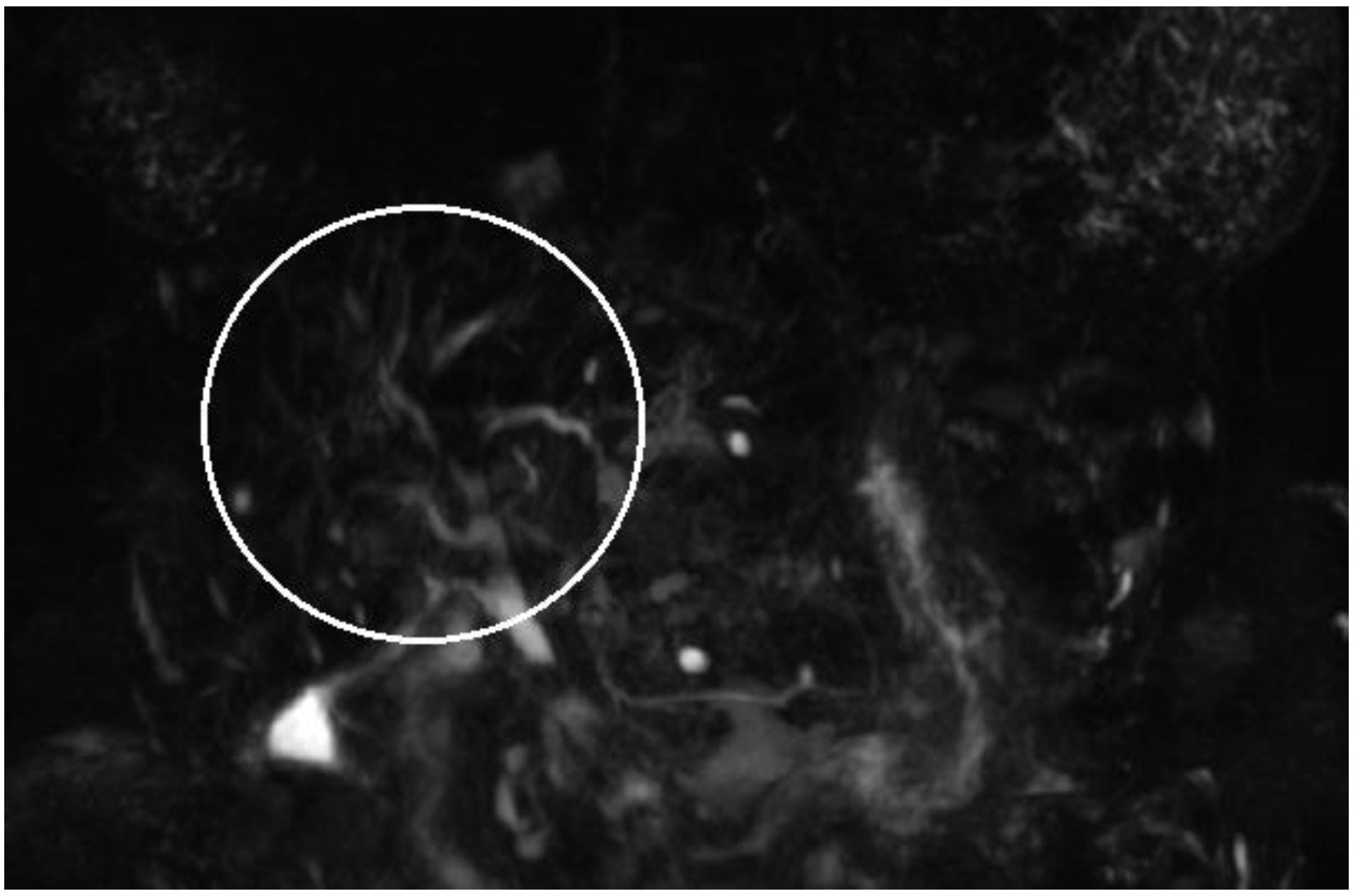

Supplement: Supplementary file 3 [file SupplementaryFile3.pdf]
