# Supplementary material for: Case Series: Severe cholangitis as an immune checkpoint inhibitor induced adverse event in four cases: management and treatment options including tocilizumab and ursodeoxycholic acid
Source: Front Immunol. 2026 Oct 27;17:1929147. doi: 10.3389/fimmu.2026.1929147 (PMC13756901; doi:10.3389/fimmu.2026.1929147)

Figure S4

Liver biopsy, immunohistochemistry using anti CD8; original magnification: 40

CD8 positive lymphocytes (A) compared with the fewer CD20 positive lymphocytes (B) (inset lower left).

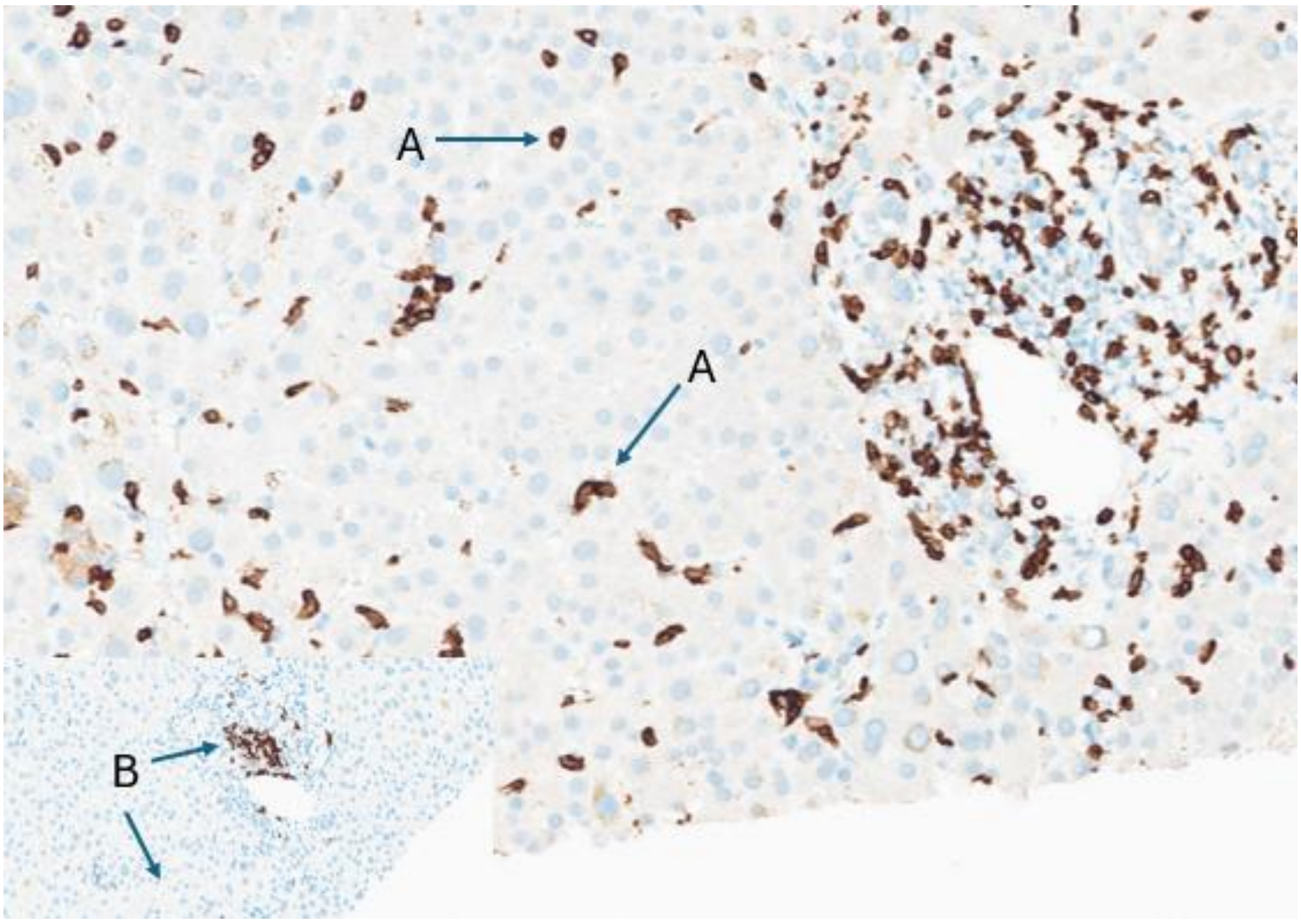

Supplement: Supplementary file 4 [file SupplementaryFile4.pdf]
