# Supplementary material for: Case Series: Severe cholangitis as an immune checkpoint inhibitor induced adverse event in four cases: management and treatment options including tocilizumab and ursodeoxycholic acid
Source: Front Immunol. 2026 Oct 27;17:1929147. doi: 10.3389/fimmu.2026.1929147 (PMC13756901; doi:10.3389/fimmu.2026.1929147)

Figure S5a, b

Radial MRCP and coronal section contrast-enhanced CT in the portal-venous phase demonstrating a distal CBD stricture (arrow) with diffuse wall thickening and upstream intrahepatic duct dilatation.

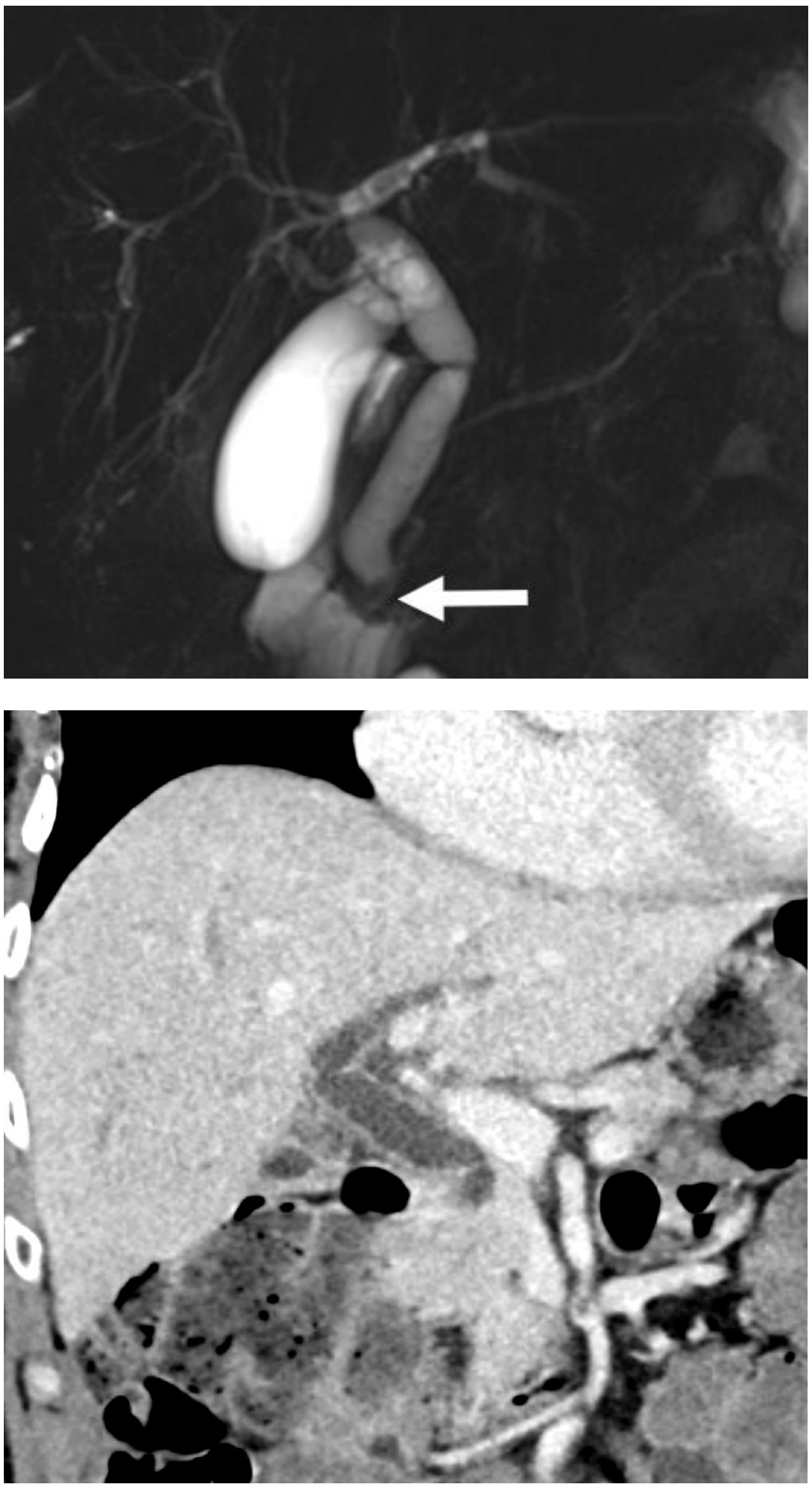

Supplement: Supplementary file 5 [file SupplementaryFile5.pdf]
