# Supplementary material for: Case Series: Severe cholangitis as an immune checkpoint inhibitor induced adverse event in four cases: management and treatment options including tocilizumab and ursodeoxycholic acid
Source: Front Immunol. 2026 Oct 27;17:1929147. doi: 10.3389/fimmu.2026.1929147 (PMC13756901; doi:10.3389/fimmu.2026.1929147)
Supplement: Supplementary file 6 [file SupplementaryFile6.pdf]

Figure S6a, b

PET CT. Common hepatic duct with moderate dilatation and with intense abnormal FDG accumulation throughout most of its length (first two images); mild generalised increased gallbladder mural uptake (second two pictures). Pembrolizumab induced. Patient asymptomatic, liver tests including bilirubin normal.

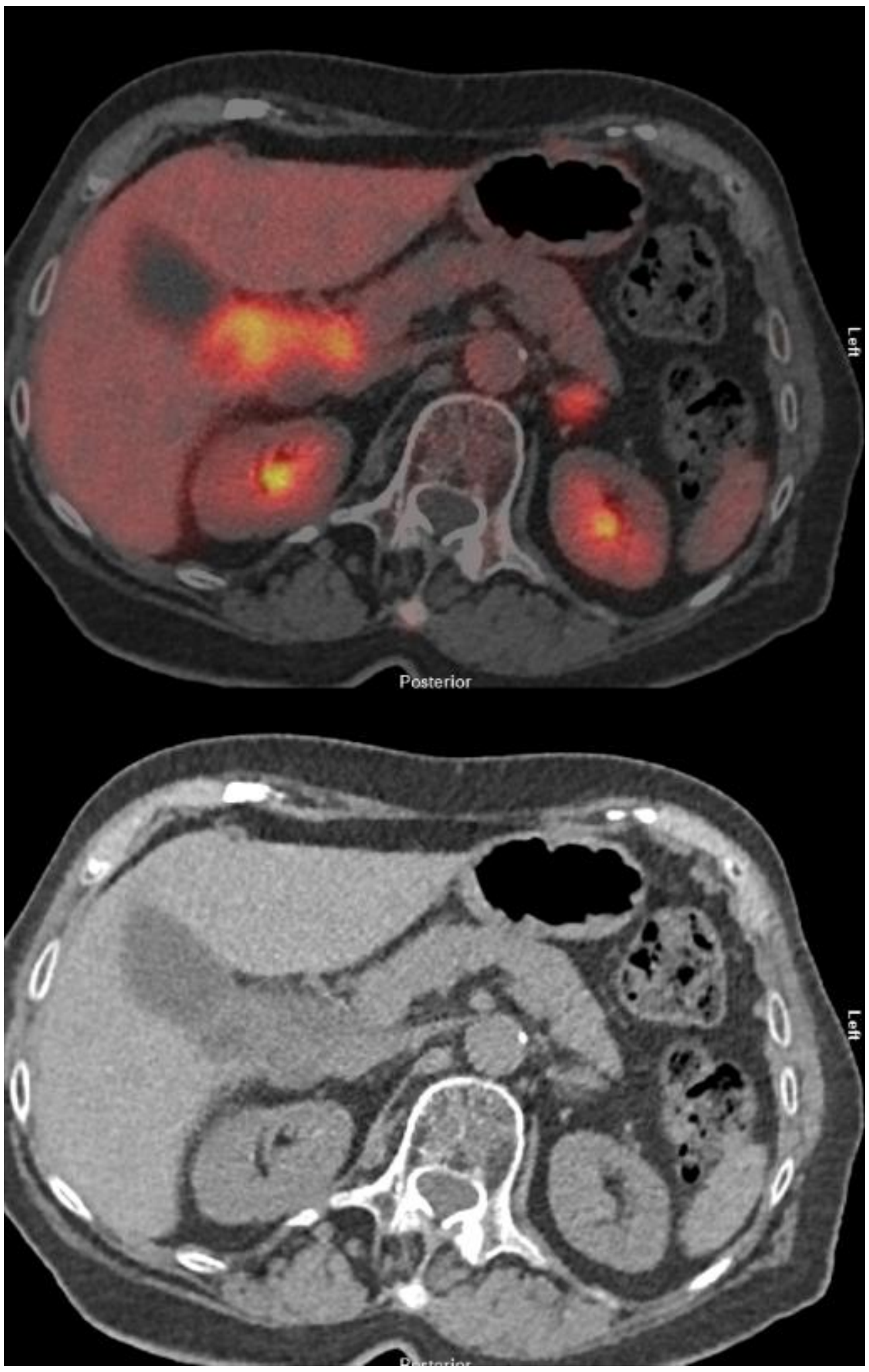

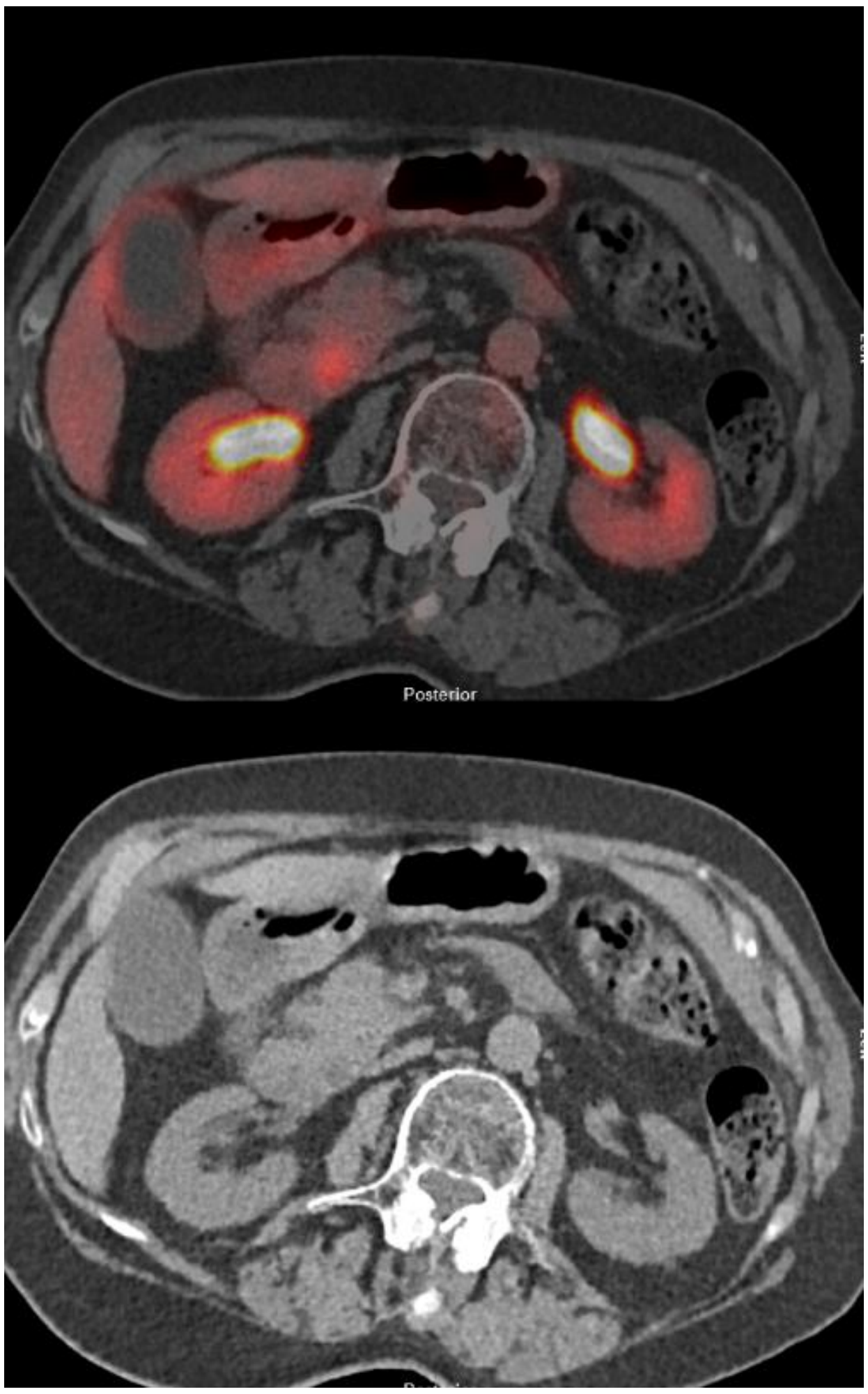
Posterior
