# Supplementary material for: Case Series: Severe cholangitis as an immune checkpoint inhibitor induced adverse event in four cases: management and treatment options including tocilizumab and ursodeoxycholic acid
Source: Front Immunol. 2026 Oct 27;17:1929147. doi: 10.3389/fimmu.2026.1929147 (PMC13756901; doi:10.3389/fimmu.2026.1929147)
Supplement: Supplementary file 7 [file SupplementaryFile7.pdf]

Table S1: summarizes management of ICI-C.

**History: treatment with ICIs ?**	**Yes, predominantly with anti-PD1**
**Laboratory findings**	• Biochemistry: cholestatic or mixed liver injury typically<br>• Autoimmune profile – typically negative<br>• Infections: HAV, HBV, HCV, HEV, CMV, EBV to be excluded
**Past medical history**	• Alcohol?<br>• Another drug induced liver injury?<br>• Another preexisting liver disease?
**Imaging: US, CT with intravenous contrast**	• Assess possible liver involvement by the tumour<br>• Assess for evidence of cholangitis<br>• Exclude Budd-Chiari syndrome<br>• Exclude (ICI induced) pancreatitis
**Imaging: MRCP, MRI liver with contrast, EUS**	Evaluate extra- and intrahepatic biliary system<br>Features typical for ICI-C<br>• Intrahepatic and/or extrahepatic biliary dilatation with no obvious biliary obstruction (stones / tumour)<br>• Stricturing and irregularity of intrahepatic biliary ducts<br>• Biliary duct wall thickening<br>• Ductal and periductal enhancement, diffusion restriction
**Urgent liver biopsy (percutaneous or trans-jugular)**	If all above negative and high suspicion of ICI-C (including in those with a rather hepatocellular liver injury in laboratory tests)
**ERCP / biliary drainage in general**	Only if co-incidental pathology present:<br>• Stones / sludge<br>• Extrahepatic biliary obstruction by the tumour or enlarged lymph-nodes
**Treatment**	• Cessation of immunotherapy (if liver injury > 3x ULN)<br>• Review medication and discontinue if hepatotoxic potentially<br>• Glucocorticosteroids orally / intravenously<br>• Ursodeoxycholic acid – always<br><br>**If refractory to glucocorticosteroids (observed in majority cases)**<br>• Tocilizumab – in more severe cases<br>• Mycophenolate Mofetil<br>• Azathioprine<br>• Tacrolimus

Table 1
